# Synthesis and Characterization of Polymers Containing Ethynylene and Ethynylene-Thiophene Based Alternating Polymers Containing 2,1,3-Linked Naphthothiadiazole Units as Acceptor Linked with Fluorine as Donor: Electrochemical and Spectroscopic Studies

**DOI:** 10.3390/polym14194139

**Published:** 2022-10-03

**Authors:** Ahmed G. S. Al-Azzawi, Elham M. A. Dannoun, Shujahadeen B. Aziz, Ahmed Iraqi, Sameerah I. Al-Saeedi, Muaffaq M. Nofal, Ary R. Murad

**Affiliations:** 1 Department of Chemistry, University of Sheffield, Sheffield S3 7HF, UK; 2 Department of Chemistry, College of Education for Pure Science, University of Mosul, Mosul 41002, Iraq; 3 Department of Mathematics and Science, Woman Campus, Prince Sultan University, P.O. Box 66833, Riyadh 11586, Saudi Arabia; 4 Hameed Majid Advanced Polymeric Materials Research Laboratory, Department of Physics, College of Science, University of Sulaimani, Qlyasan Street, Sulaimani 46001, Iraq; 5 The Development Center for Research and Training (DCRT), University of Human Development, Kurdistan Region of Iraq, Sulaymaniyah 46001, Iraq; 6 Department of Chemistry, College of Science, Princess Nourah bint Abdulrahman University, P.O. Box 84428, Riyadh 11671, Saudi Arabia; 7 Department of Mathematics and Science, Prince Sultan University, P.O. Box 66833, Riyadh 11586, Saudi Arabia; 8 Department of Pharmaceutical Chemistry, College of Medical and Applied Sciences, Charmo University, Chamchamal, Sulaimani 46023, Iraq

**Keywords:** conjugated polymers, XRD study, thermal analysis, optical properties, naphthothiadiazole (NT)

## Abstract

The effect of ethynylene or ethynylene–thiophene spacers on the band gap of alternating polymers, containing 4,9-naphthothiadiazole units as an acceptor and 2,7-linked fluorene repeat units as a donor, were investigated. The Sonogashira coupling reaction was employed to prepare the two novel copolymers, namely ((9,9-dioctyl-fluorene)-2,7-diethynylene-alt-4,9-2,1,3-naphthothiadiazole (PFDENT) and poly(5,5’-(9,9-dioctyl-fluorene-2,7-diyl)bis(ethynyl-2-thienyl)-alt-4,9-(2,1,3-naphthothiadiazole) (PFDTENT). The optical, electrochemical and thermal properties of the two obtained polymers were widely investigated and compared. Both resulting polymers showed low solubility in common organic solvents and moderate molecular weights. It is believed that the introduction of acetylene linkers rather than acetylene–thiophene spacers on the polymer chains reduces the steric hindrance between the donor and acceptor units which leads to the adoption of more planar structures of polymeric chains, resulting in decreased molecular weights of the resulting conjugated polymers. Thus, both ethynylene-based polymers and ethynylene–thiophene-based polymers showed red-shifted absorption maxima compared to their counterpart (thiophene-based polymer), owing to the adoption of more planar structures. Optical studies revealed that the new ethynylene and ethynylene–thiophene-based polymers displayed low band gaps compared to their thiophene analogue polymer **PFDTNT**. Both resulting polymers showed good thermal stability. X-ray diffraction (XRD) patterns of both polymers revealed that **PFDENT** and **PFDTENT** possessed an amorphous nature in solid state.

## 1. Introduction

In recent years, polymer solar cells (PSCs) have attracted increased attention due to their potential advantages over their inorganic semiconductor counterparts such as low manufacturing costs, mechanical flexibility of devices, light weight and large area of fabrication [1,2,3,4,5,6]. Semiconducting polymers have been widely studied for electronic applications such as organic light emitting diodes (OLEDs) and organic photovoltaics (OPVs) [7,8,9]. Furthermore, polymer semiconductors have been employed as a part of energy storage devices (batteries) which demonstrate efficient performance, especially in most lithium battery types [10]. The ability to design conjugated polymers with a variety of optical and electrochemical properties makes them attractive candidates for electronic device applications [11,12,13].

The chemical structure of polymers dictates their optical and electronic properties which determines their photovoltaic performance in devices [1,14]. It is well recognized that the tuning of the energy band gaps and energy levels of conjugated polymers by tailoring their chemical structure is an effective way to achieve low band gaps in polymers for high performance. One of the important approaches for the adjustment of these properties is to alternate electron-rich donor units with electron-deficient acceptor moieties over the polymer chain by copolymerization [15,16,17,18,19,20]. This strategy optimizes the intramolecular charge transfer (ICT) between the donor and acceptor building blocks, which enables the control of the band gap of the conjugated polymer by extending the π-delocalization system and increasing the rigidity of the structure of the polymer [16,19,21]. The introduction of fused aromatic rings within donor–acceptor (D–A) polymer chains is considered an effective strategy to reduce the band gap of the resulting polymers and extend their absorption spectrum; a result of enhancing the rigidity and planarity of their backbone. Consequently, the incorporation of various fused rings such as fluorene, carbazole and anthraceneover π-conjugated systems presents good performances in OLED and OPV devices [20]. The push/pull D–π–A approach has been studied by the research community, not only to construct low band gap polymers but also to adjust the highest occupied molecular orbital (HOMO) and lowest unoccupied molecular orbital (LUMO) energy levels in polymers to achieve promising performances in organic solar cells (OSCs) [19,22]. Further modulation of the energy levels of the alternating D–A polymer can be carried out through introducing solubilizing chains of varying steric bulk. The attachment of alky chains on the electron donor and/or acceptor units of an alternating conjugated polymer enhances its solubility, molecular weight and adjusts non-covalent interchain interactions [1,23,24]. Thus, the absorption of a broad part of the solar light spectrum for an alternating polymer can be observed if there is efficient ICT between donor and acceptor units along polymer chains, resulting in a reduced band gap of the polymer [19]. Moreover, it is believed that further changes in π-conjugated systems, delocalization, energy levels and band gap can be achieved using the linker units between the electron donor and acceptor moieties over the polymer chain.

Previous studies indicated that the linker effects between donor and acceptor moieties in the D–π–A system play a crucial role in determining the band gap of the polymer, as a linker can affect the rigidity and planarity of polymer chains [25,26]. The incorporation of different spacers over the polymer chains results in significant differences in electronic delocalization and rigidity of the backbone of the resulting polymers [25]. Zhang et al. compared the properties of thiophene-based polymers, which consisted of thiophene homo-polymers as well as alternating thiophene–acetylene copolymers. This study showed the considerable influence of the introduction of the acetylene linker on the solubility and optoelectronic properties of the resulting polymer. This displayed a much lower solubility and lower molecular weight compared to its analogue without acetylene linkers owing to higher rigidity and coplanar conformations of the polymer backbone [20]. However, other literature reports have found that the incorporation of the ethynylene units over polymer main chains does not have an effect on the solubility and molecular weight of the obtained polymers [25]. Du et al. also prepared a series of ethynylene–thiophene-based polymers with either phenylene or carbazole units as electron donor units and benzothiadiazole (BT) as acceptor repeat units. The different positions of the ethynylene units and thiophene units between the electron donor and acceptor units over the polymer chains exhibited differences in the optical, electronic and photovoltaic properties of these polymers. The insertion of ethynylene units between BT units and thiophene units displayed the best performance among these polymers, with a PCE of 1.6% for the carbazole-based polymer when fabricated with (6,6)-Phenyl C_71_ butyric acid methyl ester (PC_71_BM) in bulk heterojunction (BHJ) devices [27]. The incorporation of ethynylene units along the polymer backbone is a successful approach for attaining low HOMO levels in conjugated polymers and could thus potentially enhance the *V_oc_* value of devices. In addition to a high *V_oc_*, a lower HOMO level for the conjugated polymer also translates into a better oxidation stability of the polymer [27].

**Figure 1:**
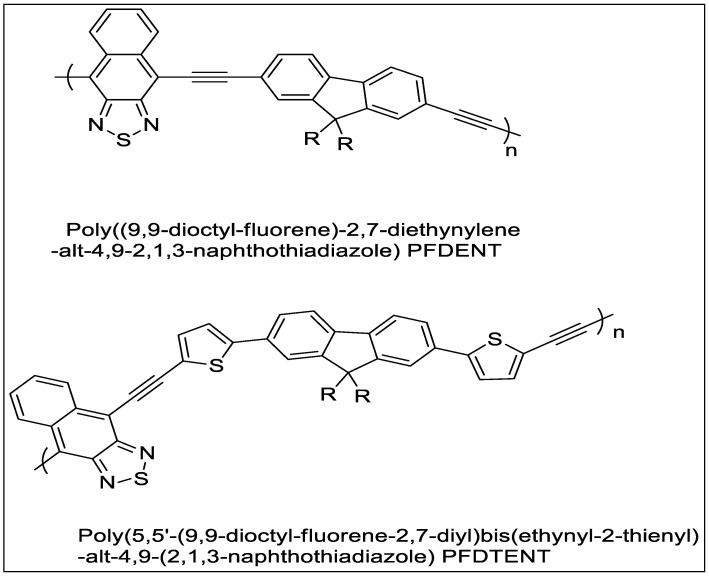
Structures of PFDENT and PFDTENT.

The main target in this study is to explore the use of ethynylene or ethynylene–thiophene spacers in alternate D–A polymers, comprising alternate naphthothiadiazole electron accepting units and alternate fluorine electron donating units. The impact of ethynylene and ethynylene–thiophene units on the optoelectronic properties of the resulting polymers will be investigated. The **PFDENT** conjugated polymer (Figure 1), which contains ethynylene units in the alternate D–A polymer backbone, was successfully synthesized using the Sonogashira coupling reaction. The ethynylene units are located between the electron-deficient units and the electron-rich units to yield a new copolymer (**PFDENT)**. In the ethynylene counterpart polymer, thiophene units are placed next to the electron donor units to be positioned between the ethynylene units and the naphthothiadiazole electron-acceptor units to yield a new polymer (**PFDTENT**). The optical, electrochemical and thermal properties of the resulting polymers were investigated and compared. The results displayed that ethynylene and thiophene-based polymers (**PFDTENT**) exhibited a lower band gap relative to its ethynylene counterparts (**PFDENT**). The polymer **PFDTENT**, which has ethynylene and thiophene linkers between electron donor and acceptor units, exhibited a red-shifted absorption spectra when compared to its counterpart (**PFDENT**). The synthesis and characterization of these **PFDENT** polymers will be discussed in this study and a comparison of its properties with its thiophene counterpart **PFDTNT**, as well as a comparison to the properties of the polymer with ethynylene-thiophene linkers **PFDTENT**, will be conducted.

## 2. Experimental Section

### 2.1. Materials

The 2,3-diaminonaphthalene (**S1**) and 2,7-dibromo-9,9-dioctylfluorene (**S2**) were purchased from Sigma Aldrich (Gillingham, United Kingdom) and used as received. All chemicals and solvents, with the exception of those stated below, were purchased commercially and used without further purification unless otherwise stated. Tetrahydrofuran (THF) was distilled and dried over sodium benzophenone under an inert nitrogen atmosphere. Toluene was dried and distilled over sodium under an inert atmosphere. Acetonitrile was dried and distilled over phosphorus pentoxide under an inert argon atmosphere, then stored over molecular sieves (3 Å).

### 2.2. Measurements

Both ^1^H and ^13^C nuclear magnetic resonance (NMR) spectra were conducted by using the Bruker Advance (400 MHz) NMR spectrometer at room temperature using deuterated chloroform or dichloromethane with tetramethylsilane (TMS) as an internal standard. The NMR shifts of all samples were described by utilization the following abbreviations terms: singlet (s), doublet (d), double doublet (dd), triplet (t), multiple (m) and broad (br). Furthermore, coupling constants (*J*) were measured in Hertz (Hz) whilst the chemical shifts were recorded in part per million (ppm). Gel permeation chromatography (GPC) measurements were performed on polymer solutions using chloroform or 1,2,4-trichlorobenzene (TCB) as eluents at a flow rate of 1 mL/ min. The system of GPC was calibrated against a series of narrow polystyrene standards (polymer laboratories) mixed C columns (7.5 × 300 mm), and a guard of 7.5 × 50 mm which were thermostated with chloroform at 40 °C. A 1037 Differential Refractive Detector was used in the system as the main detector. Each sample of polymer was prepared as a solution of 2.5 mg/mL to measure the molecular weight of the sample.

Elemental Analyzer (the Perkin Elmer 2400 CHNS -Series II) was employed to measure the elemental analysis (CHNS). In this technique, the Schöniger oxygen flask combustion method was used to analyze anions such as halides and sulfur. The mass spectra of monomers were registered on a Perkin Elmer Turbomass Mass Spectrometer equipped with auto system XL GC. UV-visible absorption spectra were studied at ambient temperature using a Hitachi U-2010 Double Beam UV/Visible Spectrophotometer. Solution samples of polymers in chloroform were measured by using quartz cuvettes (optical path length = 10 mm). Thin films of the polymers were prepared for UV-visible absorption spectra measurements by dip coating quartz substrates into approximately 1 mg cm^−3^ solutions in chloroform, then drying at room temperature. Cyclic voltammograms were conducted by a Princeton Applied Research Model 263A Potentiostat/Galvanostat. Measurements of cyclic voltammetry were performed at room temperature using tetrabutyl ammonium perchlorate (TBAClO_4_) solution in acetonitrile (0.1 mol dm^−3^) as the electrolyte solution. A typical three-electrode system was used. An Ag/Ag+ reference electrode (silver wire) was placed in 0.01 mol dm^−3^ silver nitrate solution in the electrolyte solution), a platinum working electrode (platinum wire) was purged with an argon atmosphere. Polymer thin films were coated by drop casting 1.0 mm^3^ of polymer solutions in chloroform (HPLC grade) (1 mg cm^−3^) onto the working electrode (Pt disk), then dried under inert conditions. As a reference redox system, ferrocene (Fc/Fc+) was employed according to IUPAC’s recommendation [28]. Thermogravimetric analyses (TGAs) were conducted using a Perkin Elmer (TGA-1) with temperature range between 30 and 900 °C at a scan rate of 10 °C min^−1^ under inert conditions. Powder X-ray diffraction profiles of polymers were obtained using a Bruker D8 advance diffractometer with a CuK-α radiation source (1.5418 Å, rated as 1.6 kW). The scanning angle was performed over the range of 2–40°.

### 2.3. Preparation of the Monomers and Polymers

#### 2.3.1. 1,4-Dibromo-2,3-diaminonaphthalene (**1**) [29]

A solution of bromine (3.47 mL, 10.80 g, 67.50 mmol) and 90 mL of glacial acetic acid was added drop wise into a solution of 2,3-diaminonaphthalene (**S1**) (5.00 g, 31.60 mmol) in 140 mL of acetic acid under vigorous stirring at 5 °C for 0.5 h, then stirred for 4 h at room temperature. The precipitate was filtered off and washed with 50 mL of glacial acetic acid and 500 mL of 2% sodium carbonate solution, respectively. The product was obtained as a brown precipitate (**1**) (8.20 g, 26.03 mmol, 82%) then used in the next step without further purification despite the crude having a little trace of bromine.

^1^H NMR (400 MHz, CDCl_3_) (δ/ppm): 8.35 (dd, *J* = 7.0 and *J* = 3.0 Hz, 2H), 7.42 (dd, *J* = 7.0 and *J* = 3.0 Hz, 2H), 4.32 (s, 4H).

^13^C NMR (400 MHz, CDCl_3_) (δ/ppm): 135.37, 126.72, 124.80, 124.19, 102.12.

Mass (EI+): (*m*/*z*) (M^•^⁺) 313, 315, 317.

Elemental analysis calculated for C_10_H_8_Br_2_N_2_: C, 38.01; H, 2.55; Br, 50.57; N, 8.87; found: C, 38.61; H, 2.56; N, 8.54; Br, 49.43%.

#### 2.3.2. 4,9-Dibromo-2,1,3-naphthothiadiazole (**M1**) [30]

A mixture of thionyl chloride (12.7 mL, 20.8 g, 174.8 mmol), anhydrous CHCl_3_ (80 mL) and pyridine (30 mL) was added drop wise into a solution of compound (**1**) (8.10 g, 25.63 mmol) in anhydrous CHCl_3_ (280mL) with vigorous stirring in an ice water bath. After the drop wise addition, the mixture was heated to reflux overnight. Upon completion, the reaction mixture was quenched with water. The product was extracted with DCM (3 × 300 mL), washed with saturated brine (4 × 300 mL), dried (MgSO_4_) and the solvent was evaporated in vacuo. The crude product was purified using column chromatography on silica gel and eluted with toluene: petroleum ether (70: 30%). Further purification was carried out by recrystallization from ethanol to afford orange solid crystals (**M1**) (4.31 g, 12.52 mmol, 49 % yield). The product resulted in a single spot on the TLC plate (R_f_ = 0.5) (toluene: petroleum ether. 70: 30%).

^1^H NMR (400 MHz, CDCl_3_) (δ/ppm): 8.44 (dd, *J* = 7.0 and *J* = 3.0 Hz, 2H), 7.63 (dd, *J* = 7.0 Hz and *J* = 3.0 Hz, 2H).

^13^C NMR (400 MHz, CDCl_3_) (δ/ppm): 150.50, 133.26, 128.68, 127.86 and 112.64.

Mass (EI+): (*m*/*z*) (M•⁺) 342, 344, 346.

Elemental analysis calculated for C_10_H_4_Br_2_N_2_S: C, 34.91; H, 1.17; Br, 46.45; N, 8.14; S, 9.32; found: C, 34.66; H, 0.96; Br, 46.42; N, 7.90; S, 9.20%.

#### 2.3.3. 9,9-Dioctyl-2,7-[bis(2′-trimethysilyl)ethynyl)] fluorene (**2**) [31]

A solution of 2,7-dibromo-9,9-dioctylfluorene (**S2**) (1.10 g, 2.01 mmol) in dry toluene (20 mL) and diisopropylamine (6 mL) was added, degassed and stirred to two-neck 100 mL round bottom flask under inert conditions. A mixture of copper (II) iodide (CuI) (0.016 g, 5%) as co-catalyst and bis(triphenylphosphine) palladium(II) dichloride Pd(PPh_3_)_2_Cl_2_ (0.052 g, 4%) as catalyst were added to the reaction vessel. Then, the system was degassed again. After stirring for 0.5 h, another solution containing trimethylsilyl acetylene (0.43 g, 0.62 mL, 4.40 mmol, d = 0.695 g/mL) in diisopropylamine (4.0 mL, degassed) was added via syringe to the suspension under inert conditions. After addition, the suspension reaction was degassed and then refluxed overnight to give a reddish-brown suspension solution. The reaction was monitored by TLC plate to confirm the completion of the reaction. Upon completion, the mixture was cooled to ambient temperature, then the solvent was evaporated in vacuo to obtain a crude product. The resulting product was purified via chromatography over silica gel using petroleum ether as eluent to give a pure final product as white crystals **(2)** (1.10 g, 1.88 mmol, 94%). The purity of the product was verified by TLC (single spot Rf = 0.5) in petroleum ether.

^1^H NMR (400 MHz, CDCl_3_) (δ/ppm): 7.61 (d, *J* = 8.0 Hz, 2H), 7.47 (dd, *J* = 8.0 and *J* = 2.0 Hz, 2H), 7.43 (s, 2H), 1.94 (m, 4H), 1.27–1.0 (m, 20H), 0.84 (t, *J* = 7.0 Hz, 6H), 0.57–0.48 (br, 4H), 0.30 (s, 18H).

^13^C NMR (400 MHz, CDCl_3_) (δ/ppm): 150.93, 140.85, 131.22, 126.21, 121.73, 119.83, 106.07, 94.25, 55.23, 40.34, 31.79, 29.89, 29.24, 23.59, 22.60, 14.10 and 0.06.

Mass (EI+): (*m*/*z*) (M•⁺) 582, 583, 584.

Elemental analysis calculated for C_39_H_58_Si_2_: C, 80.34; H, 10.03 found; C, 79.83; H, 9.82.

#### 2.3.4. 9,9-Dioctyl -2,7-diethynylfluorene (**M2**) [31]

A tow-neck round bottom flask (50 mL) containing a solution of compound (**2**) (1.00 g, 1.71 mmol) in dry THF (15 mL) was vigorously stirred under degassed conditions. Then, the basic aqueous solution (KOH) (2.5 mL, 25%wt) in methanol (10 mL) was slowly added to the reaction vessel under an inert condition. The reaction was left at ambient temperature for 2 h, the mixture was then extracted with DCM, washed with brine and dried over (MgSO_4_). DCM was removed in vacuo to yield white-yellow crystals as pure product (**M2**) (0.72 g, 1.64 mmol, 96%). The product resulted in a single spot on the TLC plate (Rf = 0.36) in 100% hexane.

^1^H NMR (400 MHz, CDCl_3_) (δ/ppm): 7.65 (d, *J* = 8.0 Hz, 2H) 7.50 (dd, *J* = 8.0 and *J* = 2.0 Hz, 2H), 7.48 (s, 2H), 3.17 (s, 2H), 1.97–1.93 (m, 4H), 1.27–1.05 (m, 20H), 0.84(t, *J* = 7.0, 6H), 0.60–0.53 (br, 4H).

^13^C NMR (400 MHz, CDCl_3_) (δ/ppm): 151.05, 141.01, 131.25, 126.55, 120.84, 119.98, 84.53, 55.22, 40.21, 31.77, 29.94, 29.20, 23.65, 22.59 and 14.08.

Mass (EI+): (*m*/*z*) (M•⁺). 438, 439, 440.

#### 2.3.5. 2,7-Dithienyl-9,9-dioctylfluoroene (**3**) [32]

The 2,7-Dibromo-9,9-dioctylfluorene (**S2**) (0.50 g, 0.91 mmol) was dissolved in dry chlorobenzene (8 mL). Next, 2-(Tributylstannyl)thiophene (0.85 g, 0.72 mL, 2.27 mmol), Pd_2_(dba)_3_ (0) (0.042 g, 5 %) and tri(o-tolyl)phosphine (0.028 g, 10 %) were added to the solution and refluxed at 120 °C under an inert atmosphere overnight. Upon completion, the mixture was cooled to room temperature. Diethyl ether (100 mL) was added to the reaction mixture. The organic phases were combined, washed with brine (2 × 200 mL) and dried over (MgSO_4_). The solvent was removed in vacuo, the crude product was then purified via silica gel column chromatography using petroleum ether: DCM (13:1) to yield a pure product (3) as a faint blue oil material (0.46 g, 0.82 mmol, 91%).

^1^H NMR (400 MHz, CDCl_3_) (δ/ppm): 7.70 (d, *J* = 8.0 Hz, 2H), 7.63 (d, *J* = 8.0 Hz, 2H), 7.58 (s, 2H), 7.41 (d, *J* = 4.0 Hz, 2H), 7.32 (d, *J* = 5.0 Hz, 2H), 7.14 (t, *J* = 4.0 Hz, 2H), 2.06–2.00 (m, 4H), 1.28–1.04 (m, 20H), 0.81(t, *J* = 7.0, 6H), 0.73–0.64 (br, 4H).

^13^C NMR (400 MHz, CDCl_3_) (δ/ppm): 151.71, 145.17, 140.22, 133.28, 128.07, 124.98, 124.54, 122.90, 120.17, 120.09, 55.30, 40.42, 31.79, 30.00, 29.22, 29.19, 23.76, 22.60 and 14.07.

Mass (EI+): (*m*/*z*) (M•⁺) 554, 555, 556.

Elemental analysis calculated for C_37_H_46_S_2_; C, 80.09; H, 8.36, S, 11.56, found; C, 79.05; H, 8.14, S, 11.03%.

#### 2.3.6. 2,7-Bis(5-bromothiophen-2-yl)-9,9-di-n-octylfluorene (**4**) [33]

A solution of compound (**3**) (0.43 g, 0.77 mmol) in CHCl_3_ (10 mL) and glacial acetic acid (10 mL) was shielded from light and stirred. N-Bromosuccinimide (NBS) (0.27 g, 1.53 mmol, freshly purified by recrystallization from water) in CHCl_3_ (2 mL) and glacial acetic acid (2 mL) was slowly added. After addition, the mixture was refluxed at 70 °C for 2 h; the progress of reaction was monitored by TLC plate. Upon completion, water (200 mL) was added, and the organic layer was extracted with DCM (2 × 200 mL), dried (MgSO_4_) and the solvent was evaporated in vacuo. The crude product was purified via column chromatography using petroleum ether as eluent to yield the product (**4**) as a yellow sticky product (0.47 g, 0.66 mmol, 85 %). The product resulted in a single spot on the TLC plate (Rf = 0.45) in 100% hexane.

^1^H NMR (400 MHz, CDCl_3_) (δ/ppm): 7.69 (d, *J* = 8.0 Hz, 2H), 7.53 (d, *J* = 8.0Hz, 2H), 7.47 (s, 2H), 7.14 (d, *J* = 4.0 Hz, 2H), 7.07 (d, *J* = 4.0 Hz, 2H), 2.07–1.98 (m, 4H), 1.28–1.04 (m, 20H), 0.82 (t, *J* =7.0, 6H), 0.73–0.64 (br, 4H).

^13^C NMR (400 MHz, CDCl_3_) (δ/ppm): 151.84, 146.51, 140.41, 132.36, 130.88, 124.67, 123.06, 120.31, 119.80, 111.12, 55.31, 40.32, 31.80, 29.93, 29.22, 29.16, 23.72, 22.62 and 14.10.

Mass (EI+): (*m*/*z*) (M•⁺), 710, 712, 714.

Elemental analysis calculated for C_37_H_44_Br_2_S_2_; C, 62.36; H, 6.22, Br, 22.42, S, 9.00, found; C, 61.12; H, 6.14, Br, 23.61 S, 8.04%.

#### 2.3.7. [5,5′-(9,9-Dioctyl-9H-fluorene-2,7-diyl)bis(thiophene-5,2diyl))bis(ethyne-2,1-diyl)] bis(trimethylsilane) (**5**) [34]

A solution of compound (**4**) (0.40 g, 0.56 mmol), CuI (0.007 g, 5%) and Pd(PPh_3_)_2_Cl_2_ (0.015 g, 4%) in dry toluene (20 mL), followed by diisopropylamine (6 mL, degassed), were added to a single-neck flask and stirred under an inert atmosphere for 0.5 h, followed by the addition of trimethylsilyl acetylene (0.12 g, 0.17 mL, 1.23 mmol, d = 0.695 g/mL) in disopropylamine (4.0 mL). The system was degassed and refluxed at 70 °C for 24 h. The reaction was monitored by spot TLC to verify the completion of the reaction. After cooling to room temperature, the solvent was removed from the reaction mixture in vacuo to yield a crude product. The material was purified via column chromatography over silica gel using petroleum ether as the eluent to obtain the desired product (**5**) as a yellow oil product (0.37 g, 0.47 mmol, 85%).

^1^H NMR (400 MHz, CDCl_3_) (δ/ppm): 7.69 (d, *J* = 8.0 Hz, 2H), 7.57 (d, *J* = 8.0 Hz, 2H), 7.53 (s, 2H), 7.28–7.22 (m, 4H), 2.04–1.95 (m, 4H), 1.29–1.02 (m, 20H), 0.81 (t, *J* = 7.0, 6H), 0.72–0.64 (br, 4H), 0.29 (s, 18H).

^13^C NMR (400 MHz, CDCl_3_) (δ/ppm): 151.88, 146.52, 140.56, 133.67, 132.72, 124.98, 122.64, 122.10, 120.27, 120.14, 99.67, 97.79, 55.31, 40.27, 31.75, 29.91, 29.16, 29.11, 23.73, 22.57, 14.02 and 0.12.

Mass (EI+): (*m*/*z*) (M•⁺) 746, 747, 748.

Elemental analysis calculated for C_47_H_62_Si_2_S_2_; C, 75.45; H, 8.36, S, 8.58, Si, 7.52, found; C, 75.52; H, 8.24, S, 8.37%.

#### 2.3.8. 5,5′-(9,9-Dioctyl-fluorene-2,7-diyl)bis(2-ethynylthiophene) (**M3**) [34,35]

To a solution of compound (**5**) (0.30 g, 0.40 mmol) in dry THF (10 mL), an aqueous KOH (0.7 mL, 25 % wt) in methanol (4 mL) was slowly added and stirred at ambient temperature under an inert atmosphere. The mixture was degassed and left for 3 h. The reaction was monitored by TLC to confirm the completion of the reaction. Upon completion, the mixture was extracted using a DCM (3 × 100) and washed with water (2 × 100 mL). The organic layers were combined, dried (MgSO_4_) and the solvent was removed in vacuo. The desired product (**M3**) was obtained as a brown sticky product (0.22 g, 0.36 mmol, 91 %) which resulted in a single spot on the TLC plate in 100% petroleum ether.

^1^H NMR (400 MHz, CDCl_3_) (δ/ppm): 7.70 (d, *J* = 8.0 Hz, 2H), 7.59 (d, *J* = 8.0 Hz, 2H), 7.54 (s, 2H), 7.31–7.25 (m, 4H), 3.43 (s, 2H), 2.04–1.99 (br, 4H), 1.29–1.02 (m, 20H), 0.81 (t, *J* = 7.0, 6H), 0.72–0.64 (br, 4H).

^13^C NMR (400 MHz, CDCl_3_) (δ/ppm): 151.91, 146.85, 140.66, 134.21, 132.62, 125.09, 122.67, 120.84, 120.35, 120.16, 81.97, 77.23, 55.37, 40.30, 31.78, 29.93, 29.19, 29.15, 23.74, 22.60 and 14.06.

Mass (EI+): (*m*/*z*) (M•⁺) 603, 604.4, 605, 603.

Elemental analysis calculated for C_41_H_46_S_2_; C, 81.67; H, 7.69, S, 10.64, Si, 7.52, found; C, 80.68; H, 7.53, S, 10.43%.

#### 2.3.9. Poly((9,9-dioctyl-fluorene)-2,7-diethynylene-alt-4,9-2,1,3-naphthothiadiazole)**PFDENT** [36]

The **M2** (0.120 g, 0.35 mmol) and monomer **M1** (0.153 g, 0.35 mmol) were added to a single-neck 100 mL round-bottom flask and placed under argon. Dry THF (2 mL), toluene (4 mL), followed by diisopropylamine (3 mL, degassed) were added and the system was degassed. To this mixture, Pd(PPh_3_)_2_Cl_2_ (0.035 g, 0.049 mmol, 14%) and CuI (0.015 g, 0.078 mmol, 23%) were added, degassed and refluxed at 75 °C for 5 h. Upon completion, the reaction mixture was cooled to room temperature and precipitated in degassed methanol (300 mL), while the polymer solution was left to stir overnight. The solution was filtered through a membrane and cleaned using a Soxhlet extraction with different organic solvents in the following order: methanol, acetone, hexane and toluene for 24 h. The toluene fraction was concentrated to 10 mL in vacuo and the solution precipitated in degassed methanol (300 mL). The resulting precipitate was stirred overnight and the bright dark purple product collected by a membrane filter.

^1^H NMR (400 MHz, CDCl_3_) (δ/ppm): 8.78–8.71 (br, 2H), 7.92–7.65 (br, 6H), 7.92–7.65 (br, 2H), 2.23–1.96 (br, 4H), 1.31–1.01 (br, 20H), 0.89–0.57 (br, 10H).

Toluene fraction (18% yield) GPC in TCB at 140 °C (*M_n_* = 10,000), (*M_w_* = 30,200), (PDI = 3.0).

Elemental Analysis calculated for C_43_H_44_N_2_S: C, 83.18; H, 7.14; N, 4.51; S, 5.16. Found: C, 78.30; H, 6.98; N, 3.62; S, 3.38%.

#### 2.3.10. Poly(5,5’-(9,9-dioctyl-fluorene-2,7-diyl)bis(ethynyl-2-thienyl)-alt-4,9-(2,1,3-naphthothiadiazole)**PFDTENT** [36]

**PFDTENT** was prepared according to the polymerization method in **PFDENT**. A single-neck 100 mL round-bottom flask was charged with **M3** (0.180 g, 0.30 mmol) and **M1** (0.103 g, 0.30 mmol) under inert atmosphere. Dry THF (2 mL) and toluene (4 mL), followed by diisopropylamine (3 mL, degassed), were added and degassed at ambient temperature. To this mixture, Pd(PPh_3_)_2_Cl_2_ (0.029 g, 0.042 mmol, 14%) and CuI (0.013 g, 0.068 mmol, 23%) were added and the system was degassed again. The mixture was refluxed at 75 °C for 1 h. After carrying out the extraction and purification using different organic solvents, the polymer was only obtained in the toluene fraction to obtain a dark green product.

^1^H NMR (400 MHz, CDCl_3_) (δ/ppm): 8.64–8.58 (br, 2H), 7.80–7.37(br, 12H), 2.14–1.99 (br, 4H), 1.31–1.06 (br, 20H), 0.86–0.68 (br, 10H).

Toluene fraction (17% yield) GPC in TCB at 140 °C (*M_n_* = 11,500), (*M_w_* = 29,700), (PDI = 2.5).

Elemental Analysis calculated for C_51_H_48_N_2_S_3_: C, 78.02; H, 6.16; N, 3.57; S, 12.25%. Found: C, 72.91; H, 6.14; N, 2.46; S, 10.25%.

## 3. Results and Discussion

### 3.1. Synthesis and Characterization

**Scheme 1:**
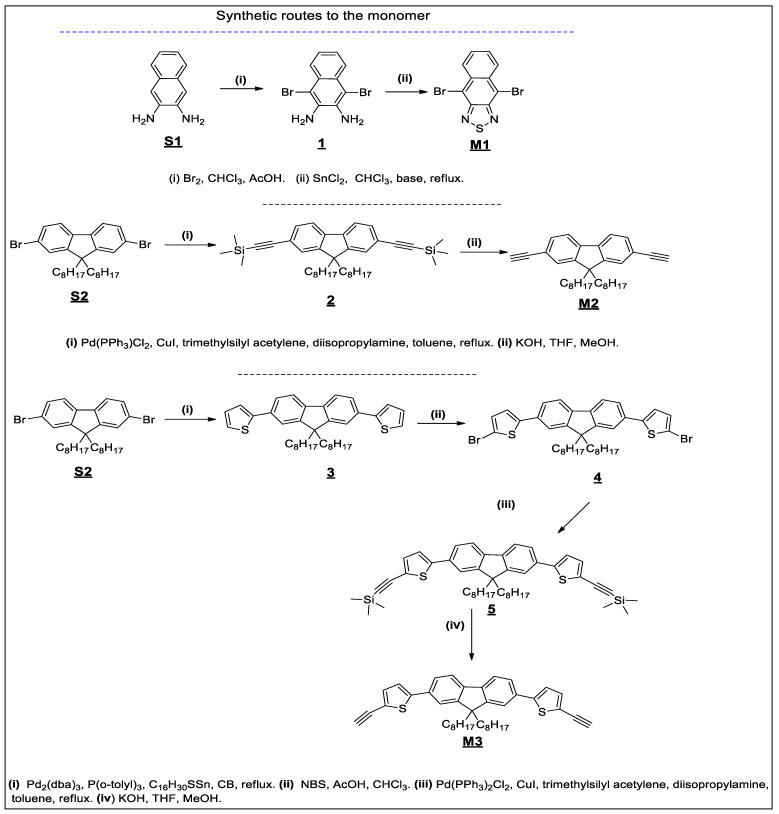
Synthetic route towards monomers (M1–M3).

The general synthetic routes to prepare the intermediate monomers **M1**–**M3** are outlined in Scheme 1. The key monomer **M1** was synthesized from 2,3-diaminonaphthalene (**S1**) by bromination with bromine using acetic acid to obtain the corresponding dibromo compound **1**, followed by ring closure using excess thionyl chloride in basic conditions, generating **M1** in a yield of 49% after recrystallization from ethanol. Moreover, the final monomer **M2** (9,9-dioctyl-2,7-diethynylfluorene) was synthesized from 9,9-dioctyl-2,7-dibromofluorene (**S2**) as starting material. This monomer requires to be kept at low temperature and in the dark before use. The first step in the monomer synthesis was a functionalization of the 2,7 positions of fluorene with trimethylsilyl acetylene using a Sonogashira reaction, which resulted in compound (**2**) (9,9-dioctyl-2,7-[bis(2′-trimethysilyl)ethynyl)]fluorene) in a yield of 85.4%. The reaction was carried out in the presence of Pd and CuI as the catalysts with diisopropylamine/(THF, toluene) as the reaction media under an inert atmosphere. The procedure is well established in the literature [31]. The final monomer **M3** can be obtained in four steps following the modified procedures [34,35]. The first step requires the use of a Stille reaction between 2-(tributylstannyl) thiophene and 2,7-dibromo-9,9-dioctylfluorene (**S2**) to obtain 2,7-dithienyl-9,9-dioctylfluorene (**3**). The reaction was carried out in chlorobenzene at 120 °C overnight using Pd_2_(dba)_3_and tri(o-tolyl)phosphine as a catalyst. In this reaction, a high boiling point solvent helps the reaction to proceed to completion. The reaction was high yielding (91%) after the purification of the product by column chromatography. The second step is a bromination reaction, and compound (**3**) was brominated at the 5,5 positions of thiophene units using NBS in a CHCl_3_/AcOH solvent mixture to obtain the desired product of 2,7-bis(5-bromothiophen-2-yl)-9,9-di-n-octylfluorene (**4**) in a high yield. Using a ratio of NBS, compound (**3**) of 1.98:1 eliminated concerns about the formation of tri and tetra brominated compounds that are difficult to be separated from the desired product. The following steps involved a Sonogashira reaction of dibromide (**4**) and trimethylsilyl acetylene using a Pd/Cu catalyst and base to obtain compound (**5**). The crude product was purified via silica gel column chromatography to obtain the desired product (**5**) in a high yield (85%). Compound (**5**) was then treated with a potassium hydroxide solution in THF at room temperature. The resulting deprotected product **M3** was extracted with DCM, washed with water and dried to obtain a brown colored product as a final pure monomer.

**Scheme 2:**
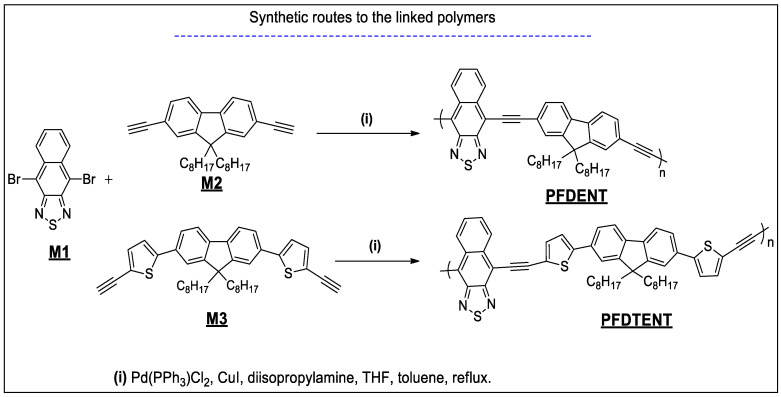
The synthetic route to polymers and their conditions and reagents.

The polymerizations of **M1** with, respectively, **M2** and **M3** by the Sonogashira reaction produced the polymers **PFDENT** (yield = 18%) and **PFDTENT** (yield = 17%) as depicted in Scheme 2. These polymerizations were easily carried out in the presence of a catalytic amount of 20% Pd(PPh_3_)_2_Cl_2_ and 14% CuI with diisopropylamine as the base media into a mixture of solvents (THF, toluene) under an inert atmosphere. Using the excess of co-catalysts as modification is required to guarantee a successful reaction. It was observed that the polymerization time varied between 1 and 5 h with large quantities of polymer precipitates forming during the reactions. Both resulting polymers were purified by successive Soxhlet extractions with different common organic solvents. It was also noted that large amounts of the resulting polymers remained as intractable materials in the Soxhlet thimbles after extraction. The low solubilities of the resulting polymers explain the low yields observed from these polymerizations. The products obtained from the toluene Soxhlet fraction (which is generally the fraction obtained from the extraction of processable materials from most conjugated polymers) were soluble in common organic solvents. The chemical structures of these copolymers were verified by ^1^H NMR and elemental analysis. Details of the synthesis of the monomers **M1–M3**, the polymers and their characterizations are described in the experimental section.

GPC analysis of these polymers evaluated the number and weight-average molecular weights (*M_n_* and *M_w_*) using a TCB as the eluent at 140 °C (Table 1). The GPC measurements of the ethynylene-based polymer (**PFDENT**) and the ethynylene–thiophene-based polymer (**PFDTENT**) exhibited moderate molecular weights. These polymers were only obtained in their toluene fractions. It was speculated that the incorporation of the ethynylene and ethynylene–thiophene spacers over the polymer chains results in a decrease in the torsion angle between alternate units along polymer chains, leading to the aggregation of polymer chains as a result of their coplanar geometry [37,38]. This then limits the accessibility to soluble high molecular weight polymers. Despite most of **PFDENT** and **PFDTENT** being extracted in the toluene fractions, the polymers still displayed poor solubility with other organic solvents at high temperature. For comparison purposes, the ethynylene-based polymer displayed a significant reduction in molecular weight relative to its thiophene analogue polymer **PFDTNT**, which was previously synthesized and investigated by the Iraqi group [39], and where the thiophene units are replaced by ethynylene units. For example, GPC analysis estimated the *M_n_* and *M_w_* of **PFDENT** to be 10,000 and 30,200 Da, respectively. Meanwhile, the *M_n_* and *M_w_* of its counterpart **PFDTNT** are 41,100 and 79,500 Da, respectively. It is hypothesized that the presence of ethynylene-spacers facilitates π–π interchain stacking and planarization, leading to reduced *M_n_* and *M_w_* values obtained for **PFDENT** relative to those of its counterpart **PFDTNT** [38].

### 3.2. Optical Properties

**Figure 2:**
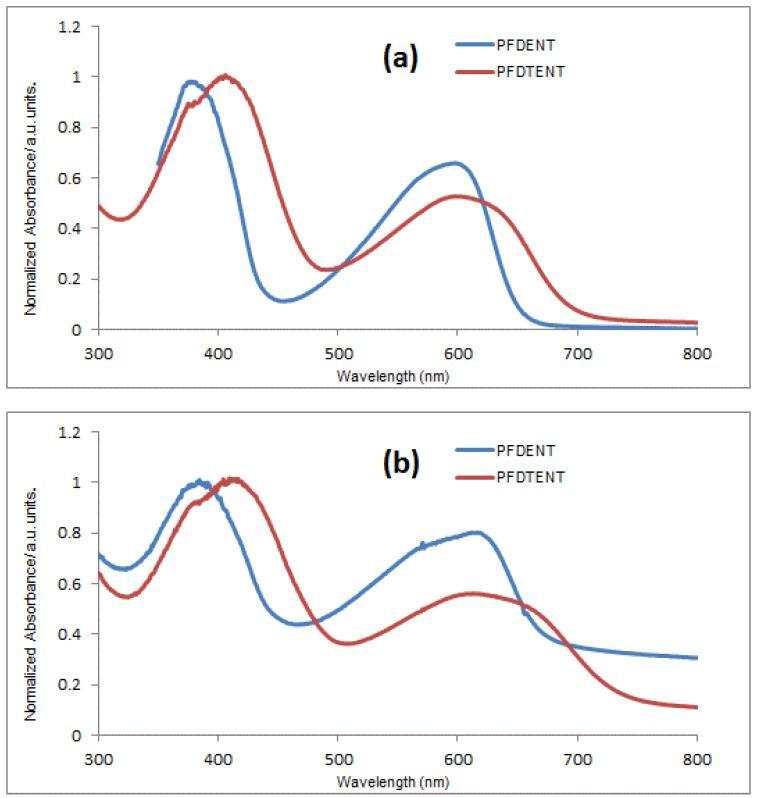
(**a**) Normalized absorption spectra of polymers in chloroform solution, (**b**) Absorption spectra of polymers as thin films.

The optical properties of the polymers were measured in dilute chloroform solutions, as seen in Figure 2a, and in thin films. The optical spectra in the solid state are presented in Figure 2b. The results for both **PFDENT** and **PFDTENT** are presented in Table 2. All resulting polymers displayed the same absorption pattern in their spectra. It can be seen that UV-vis absorption spectra of the polymers in solid state exhibit two main distinct absorption bands in the region, namely 300–410 nm and 460–700 nm. The shorter wavelength absorption peaks correspond to π–π̽ transitions of the conjugated structure of polymers. Meanwhile, the absorption bands at longer wavelengths can be attributed to ICT bands between the donor and acceptor moieties along the D–A polymer backbones. Dilute solutions of the ethylene-based polymer **PFDENT** (Figure 2a) displayed ICT absorption bands at 585. When fabricated in films (Figure 2b), the band is red-shifted and observed at 608. The bathochromic shift is mostly attributed to the ordered packing of polymer chains as well as their aggregation in solid state [40,41]. To ascertain the effect of ethynylene units on the optical properties of polymers, a further comparison was conducted between the ethynylene-based polymer **PFDENT** and its thiophene counterpart **PFDTNT** [39]. The intensity of the ICT bands of **PFDENT** is more pronounced relative to its thiophene equivalent polymer, suggesting that the incorporation of acetylene spacers instead of thiophene repeat units next to the NT units reduces the steric hindrance between these and adjacent units and enhances intramolecular charge transfer and π-overlap as well as the planarity of the polymer chains. As a result, the absorption maxima for **PFDENT** is red-shifted compared to its analogue **PFDTNT** [39]. This may extend the absorption spectrum of the synthesized polymer, although the presence of acetylene units may localize the π-electron wave function on ethynylene spacers; therefore, this decreases the electronic delocalization between the electron acceptor and the donor segments [25,38].

The optical band gap of the ethynylene-based polymer **PFDENT** was calculated to be 1.83 eV. This band gap value is wider than its thiophene analogue **PFDTNT** (1.76 eV), despite having broader absorption spectra. The incorporation of acetylene units in the fluorene-based alternate polymer (**PFDENT**) displayed red-shifted absorption maxima compared to its counterpart polymer, **PFDTNT**. However, this does not result in a lower optical band gap. This can be attributed to the presence of acetylene units in the polymer chains, which adopt ordered structures and show sharper absorption peaks owing to the improved electronic delocalization.

The absorption maxima of **PFDTENT** in solid state (Figure 2b) is located at 614 nm. The red-shifted spectra from solution to solid state can be attributed to more planar structures in films. The optical band gap (E_g_) of **PFDTENT** from the spectra is estimated to be 1.71 eV.

The ethynylene-thiophene based polymer **PFDTENT** displayed red-shifted absorption maxima and reduced band gaps, relative to its respective analogue **PFDENT**. It is speculated that the absorption maxima of **PFDTENT**, which has ethynylene–thiophene linkers, is higher than its ethynylene counterpart owing to the incorporation of additional thiophene units in the polymer chains. The additional electron-rich thiophene unit leads to overall stronger electron donating segments in **PFDTENT** compared to **PFDENT,** and as a result of stronger ICT along the polymer chains resulting in reduced band gaps. A recent study by Ozel et al. has shown that the incorporation of phenylene–acetylene units as spacers in D–A polymers does not add any extension of π-conjugation over polymer chains, suggesting that the electron-donating properties of thiophene are key in leading to the reduction in the band gaps of **PFDTENT** compared to **PFDENT** [42].

The absorption coefficients of all the resulting polymers (**PFDENT** and **PFDTENT**) in chloroform solutions are estimated to be 2.9 × 10^4^ and 3.0 × 10^4^, respectively. All data are presented in Figure 2b. Clearly, the molar absorption of polymer with ethynylene–thiophene units is slightly higher compared to their ethynylene counterparts. This is likely because the electron-donating ability of donor moieties into the polymer **PFDTENT** is stronger than its corresponding polymer without thiophene units **PFDENT**.

### 3.3. Electrochemical Properties

**Figure 3:**
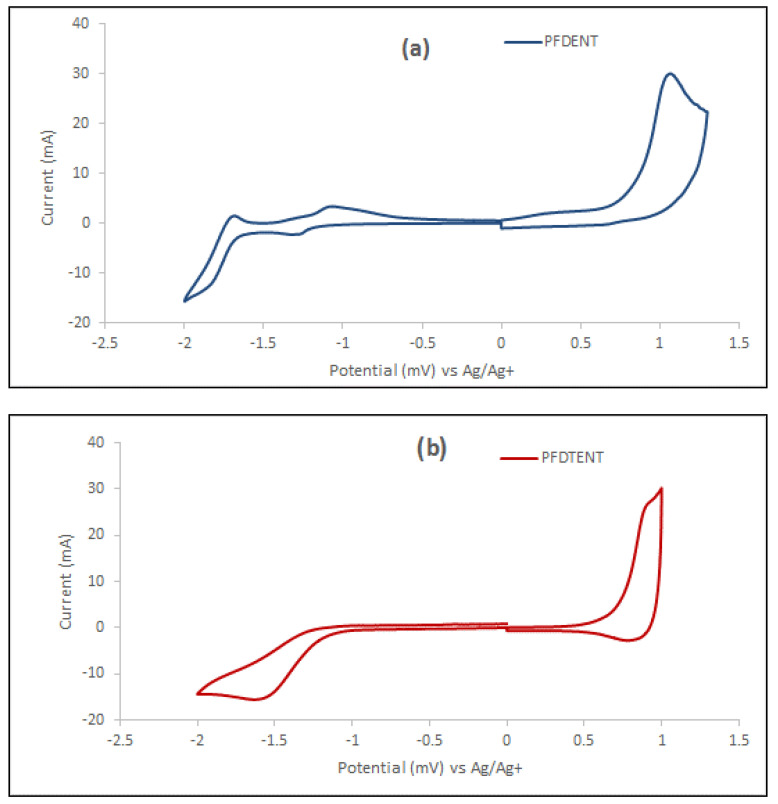
Cyclic voltammograms of (**a**) **PFDENT** and (**b**) **PFDTENT**.

The electrochemical behavior of the polymers was investigated by cyclic voltammetry (CV). The measurements were conducted using drop-cast polymer films in acetonitrile solutions with tetrabutylammonium perchlorate as an electrolyte. The cyclic voltammograms are presented in Figure 3a,b. They show that most polymers present irreversible oxidation peaks and reversible reduction potential peaks. Details of the measurements are given in the experimental section. The frontier energy levels (vs. vacuum) of the polymers were calculated from their first onset of oxidation and reduction waves as summarized in Table 2.

The potential of first onset of oxidation wave was estimated to be at 0.86 for **PFDENT**, which corresponds to the HOMO energy level of −5.58. The onset of reduction potential for **PFDENT** was observed at −1.13, which corresponds to the LUMO energy level of −3.59 eV.

A comparison of the electrochemical properties of the ethynylene-based polymer **PFDENT** to its corresponding thiophene-based polymer **PFDTNT** [39] revealed that the ethynylene-based polymer has a lower lying HOMO energy level relative to the thiophene-based polymer. Indeed, the thiophene-based polymer (**PFDTNT)** displayed a HOMO/LUMO level of −5.36/−3.50 eV. As observed, for example, by comparing **PFDENT** and its thiophene analogue **PFDTNT**, the HOMO of the ethynylene polymer **PFDENT** is −5.58 eV while the HOMO of it thiophene counterpart, **PFDTNT,** is −5.36 eV. It is hypothesized that the acetylene linkers on polymer chains in **PFDENT**, which incorporate sp-hybridized carbons, have a weakly electron withdrawing character when compared to the electron-rich thiophene units on the polymer chains in **PFDTNT**, which explains the shallower HOMO energy level of the thiophene-based polymer. This is consistent with previous literature reports that have shown decreased HOMO and LUMO energy levels with the incorporation of acetylene units over the polymer main chain [27]. These findings indicate that the incorporation of ethynylene units induces intermolecular interactions and increases the planarity of the polymer chains while changing the electronic properties of this resulting polymer and increasing its electrochemical energy band gap.

The frontier HOMO energy level (vs. vacuum) of **PFDTENT**, as calculated from the onset of oxidation wave, was estimated to be at −5.46 eV, while the corresponding LUMO energy level was determined to be at −3.60 eV. A comparison of the electrochemical properties (**Table 2**) of the ethynylene–thiophene-based polymer (**PFDTENT**) with its corresponding ethynylene polymer (**PFDENT**) reveals a considerable lowering of the HOMO energy levels of the ethynylene–thiophene-based polymer, resulting in lower band gap polymers when compared to their ethynylene counterparts. It is believed that the incorporation of thiophene units as electron-donating units in the main chain of a conjugated polymer is responsible for lifting the HOMO energy level of the resulting polymer (**PFDTENT)**, upon increasing the electron-donating ability of the donor segments on the polymer chains. Eventually, this leads to the decreased band gap polymer. As an example, the HOMO energy level of the ethynylene–thiophene-based polymer **PFDTENT** is −5.46 eV, while its ethynylene counterpart has a HOMO energy level of −5.58 eV. These results indicate the ways in which the combined thiophene units may increase the electron-donating ability on donor moieties of polymer chains, leading to change the positions of the HOMO energy levels.

### 3.4. Thermal Properties

**Figure 4:**
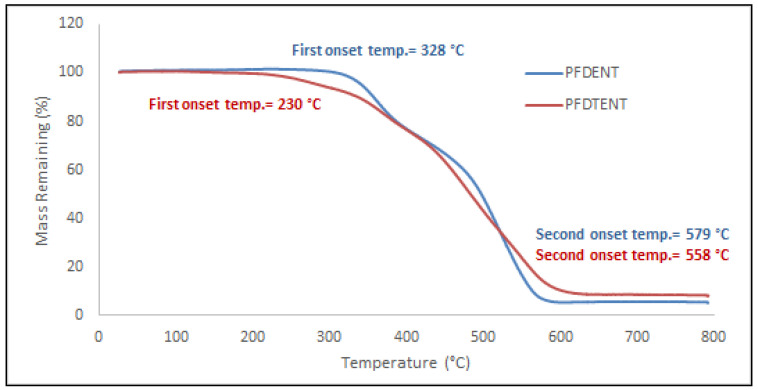
TGA plots of the resulting polymers with a heating rate of 10 °C/min under N_2_.

The thermal properties of the resulting polymers were measured by thermogravimetric analysis (TGA) (**Figure 4**). The TGA curves (a) indicate that the ethynylene-based polymer has thermal stability with decomposition temperatures, defined as the 5% weight-loss temperature, T_d_ above 300 °C in a N_2_ atmosphere at a heating rate of 10 °C/min, suggesting their excellent thermal stability. The first onset of thermal degradation temperatures for **PFDENT** was determined to be at 328 °C. The first weight loss in the degradation can be attributed to the elimination of alkyl substituents from the electron-donor moietie (fluorene) in the conjugated polymer **PFDENT**. Above 500 °C, the degradation of the polymer chains is followed by a total weight loss of 94% for **PFDENT**, which is observed when the temperature is above 590 °C. This result indicates that **PFDENT** exhibited two main-step degradation processes.

These findings indicate that the thermal stabilities of the ethynylene-based polymer are adequate for its application in solar cells and other electronic devices. It can be noted that the synthesized thiophene-based polymer **PFDTNT** [39] exhibited a higher decomposition temperature (~400 °C) relative to its ethynylene counterpart **PFDENT**. It is speculated that the high molecular weight of thiophene-based polymers (**PFDTNT**) decreases the molecular mobility and promotes chain entanglements, resulting in the increased degradation temperature of polymer. These results are consistent with previous studies that show the relationship between the molecular weight of polymers and degradation kinetics [43].

Figure 4 also shows the TGA curve of the thermal degradation of the ethynylene–thiophene-based polymer (**PFDTENT**). TGA analysis of this polymer revealed that it displays moderate thermal stabilities with a decomposition temperature (T_d_) above 230 °C. The T_d_ value was determined to be at 329 °C. The first step in the degradation is observed to range from 300 °C to 550 °C, corresponding to the elimination of alkyl chains from the electron-donor moieties of the polymer. The decomposition of the polymer chains is followed by a total loss of 94% that is observed when the temperature is above 550 °C.

The thermal results of synthesized polymer confirm that he ethynylene–thiophene-based polymer possesses good thermal properties. Compared to the ethynylene counterparts, the ethynylene-based polymer undergoes degradation at higher temperatures compared to its ethynylene–thiophene counterpart. No further comparison can be drawn between the ethynylene polymer and its analogue due to a lack of thermal analysis reported.

### 3.5. Powder X-ray Diffraction Studies

**Figure 5:**
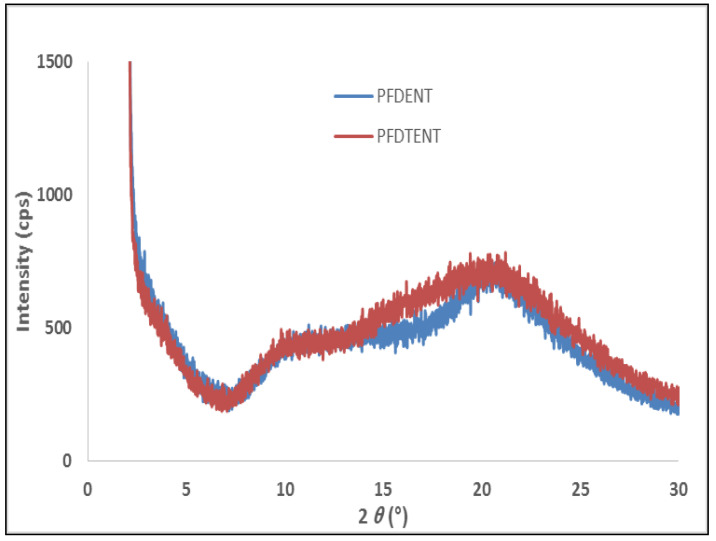
Powder XRD profiles of the target polymers.

Figure 5 displays powder X-ray diffractions (XRDs) of all the resulting polymers. The XRD profile was studied to examine the molecular packing of polymer chains. PFDENT displayed a broad peak in the wide-angle region at 2θ values of 19.95°, corresponding to a distance of 4.45. This broad peak in this area revealed that the ethynylene-based polymer adopts an amorphous structure in solid states. Generally, the results obtained are consistent with previous results in the literature concerning the effect of ethynylene spacers on the packing of polymer chains [44]. Moreover, it is interesting to compare the XRD studies of the ethynylene-based polymer **PFDENT** and its thiophene analogue polymer **PFDTNT**, which were prepared by the Iraqi group [39]. **PFDTNT** did not show any pronounced diffraction peaks in this wide-angle region compared to **PFDENT**. It is speculated that the incorporation of acetylene units along the main chains of polymer with the same system slightly promote the packing of polymer chains by improving the coplanar interactions owing to a decreased torsion angle between the electron donor and acceptor moieties.

The powder XRD pattern of the ethynylene–thiophene-based polymer revealed that the broad peak of **PFDTENT** appeared at values of 20.51°. A comparison with the ethynylene analogue polymer synthesized **PFDENT** indicates little change in the XRD pattern. However, the ethynylene counterpart polymer shows more pronounced peaks in the wide-angle region compared to the thiophene–ethylene-based polymers. It is assumed that the incorporation of thiophene spacers with ethynylene results in an increased torsion angle and therefore, an indication of the polymer amorphous conformations. Unfortunately, a further comparison cannot be drawn owing to a lack of XRD studies reported as supporting evidence.

## 4. Conclusions

In this work, two types of acetylene-based polymers were synthesized using Sonogashira coupling reactions. The first type of conjugated polymer **PFDENT** contains ethynylene units positioned between 2,1,3 naphthothiadiazole (NT) repeat units as electron-acceptor moieties and 2,7-linked fluorene alternate units as electron-donor moieties. The optical, electrochemical, thermal and chemical structures were investigated. The ethynylene-based polymer was obtained in low yield as it has low solubilities in common organic solvents. This can tentatively attribute the low solubility to the incorporation of acetylene spacers over the polymer main chains, which results in the aggregation of polymer chains and the formation of a much more rigid polymer. The HOMO/LUMO frontier energy levels of **PFDENT** were estimated to be −5.58/−3.59 eV. The properties of this polymer were compared with its corresponding thiophene polymer **PFDTNT**, which was synthesized by the Iraqi group, and which has thiophene linkers between the NT repeat units and donor units to ascertain the effects of the acetylene units on the properties of the resulting polymer. The UV-visible absorption spectroscopy revealed that the ICT band of **PFDENT** is more pronounced and red-shifted when compared to its thiophene analogue polymer **PFDTNT.** It is believed that the insertion of acetylene π-linkers along the D–A polymers reduces the steric hindrance between electron-accepting naphthothiadiazole repeat units and electron-donor repeat units. This increases its aggregation and planarity by promoting π–π interactions. Despite the ethynylene-based polymer exhibiting a red-shifted and vibrational structure compared to its thiophene counterpart, the optical band gap of this polymer is larger. This is attributed to the electronic effect of ethynylene spacers as weak electron withdrawing units, which may decrease the pull–push character and ICT of the polymer chains. The HOMO energy level of the ethynylene-based polymer (**PFDENT**) is deeper compared to its synthesized thiophene analogous polymer (**PFDTNT**). It is ascribed that the incorporation of ethynylene units as weak electron-acceptor units in the polymer chains instead of thiophene as electron-donating units results in lowering the HOMO energy level of the resulting polymer; a result of decreasing the electron-donating ability of donor units along the polymer chains. The thermal properties were studied via TGA and showed that the ethynylene-based polymer possesses good thermal stability with a decomposition temperature above 300 °C. X-ray diffraction studies revealed that **PFDENT** exhibits a certain degree of molecular ordering as a result of π–π stacking.

The second type of polymer includes ethynylene–thiophene spacers between 2,1,3 naphthothiadiazole (NT) electron-accepting units and functionalized-fluorene electron-donor alternate moieties. The resulting polymer was obtained in low yields as it has low solubilities and, as result, moderate molecular weights, which are comparable in value to their ethynylene counterpart polymer. The optical band gap of **PFDTENT** is lower compared to its corresponding ethynylene counterpart **PFDENT**. It is hypothesized that this is a result of the incorporation of additional thiophene as electron donor units over the main chains of polymer, which increases delocalization and the donating ability of the donor segments on the resulting polymer, resulting in the reduced band gap of the polymer. The TGA measurement revealed that the ethynylene–thiophene-based polymer has a low onset of decomposition compared to its ethynylene counterpart. X-ray diffraction patterns revealed that **PFDTENT** showed broad and weak peaks in the range of 15–25°, an indication that the polymer possessed an amorphous structure in solid state. Further investigations into the use of these new classes of polymers in BHJs are currently underway.

## Figures and Tables

**Table 1:** GPC data of **PFDENT** and **PFDTENT**.

Polymer	Fraction	Yield %	*M_n_* ^a^	*M_w_* ^a^	PDI ^b^
PFDENT	Toluene	18	10,000	30,200	3.0
PFDTENT	Toluene	17	11,500	29,700	2.5

^a^ Measurements conducted on the toluene and chloroform fractions of polymers using a differential refractive index (DRI) detection method. ^b^ Polydispersity index. PDI is defined by the ratio of the weight-average molar mass (*M_w_*) and the number-average molar mass (*M_n_*). It can be calculated by following equation: PDI = *M_w_*/*M_n_*.

**Table 2:** A summary of absorption in solution and in films and HOMO and LUMO levels of the polymers.

Polymer	λ_max_ Solution (nm)	ε ^a^ (M^−1^ cm^−1^)	λ_max_ Film (nm)	E_gopt_^b^ (eV)	HOMO ^c^ (eV)	LUMO ^d^ (eV)
PFDENT	585	29,600	608	1.83	−5.58	−3.59
PFDTENT	590	30,800	614	1.71	−5.46	−3.60

^a^ Absorption coefficient measured at λ_max_ = 375 nm for **PFDENT**, λ_max_ = 402 nm for **PFDTENT** in chloroform solutions. ^b^ Optical band gaps determined from the onset of UV-vis absorption spectra on solid films. ^c^ HOMO position (vs. vacuum) determined from the onset of oxidation. ^d^ LUMO position (vs. vacuum) determined from the onset of reduction.

## Data Availability

Not applicable.
